# U. S. V. M. A.

**Published:** 1895-08

**Authors:** 


### U. S. V. M. A.

SEVERAL of Long Island's veterinarians will be among the party to Des Moines in September next. Among those who have indicated their intention of joining the party will be Dr. R. A. McLean, of Brooklyn.

ANOTHER excellent article has been promised for the coming meeting of the Association by Professor M. H. Reynolds, of Minnesota, on "Hypodermic Cathartics." This will be of great interest, inasmuch as a great deal of experimental work has been done in conjunction with its preparation.

DR. JOHN M. PARKER, Haverhill, Mass., has accepted the appointment of Assistant State Secretary for Massachusetts, *vice* Dr. James B. Paige, resigned.

THE Des Moines meeting promises to take on a practical aspect that should attract a large attendance from the general practitioners of the country. The latest acquisition to the programme is a paper on "The Therapeutics of Colic," by Professor W. L. Williams, of Bozeman, Montana.

ONE of the suggestions recently made for the coming meeting is to increase the limit of time for discussion of papers. To do this the reports of State Secretaries will have to be given a time-limit. It will be just as necessary that the sessions convene promptly at 10 A.M. each day.

MCKILLIP VETERINARY COLLEGE steps to the front in her wishes to make the meeting at Des Moines a pleasant one by extending a cordial invitation to the delegates from the East to stop on their way going or returning, and visit the new college and acquaint themselves with the facilities and so forth of the school. Dean Schwarzkopf proposes to provide a luncheon for the visitors. Efforts will at once be made to arrange for this added pleasure by the special party now arranging to leave Philadelphia over the Baltimore and Ohio Railroad.

AMONG other members of the profession who contemplate attending the meeting at Des Moines are Professor W. L. Zuill, of Philadelphia, and Dr. E. Mayhew Michener, of North Wales, Pa.

MINNESOTA promises to be well represented at the coming meeting of the Association. The twin cities of St. Paul and Minneapolis will be well represented through the efforts of State Secretary Reynolds.

A VERY large attendance of veterinarians from Kansas City and vicinity is expected at the meeting at Des Moines. This should encourage other centres.

AMONG those who expect to be present from Buffalo, N. Y., will be the very efficient State Secretary, Dr. Nelson P. Hinkley.

DR. M. STALKER has accepted an appointment on the local committee of arrangements for the meeting at Des Moines.

CHAIRMAN CARY reports that the work of preparing matters connected with the Committee on Intelligence and Education are being pushed steadily along certain well-defined lines, and that the work will be well in hand by the time of meeting at Des Moines.

---

### ASSOCIATION OF VETERINARY FACULTIES OF NORTH AMERICA.

The annual meeting will be held on the first two evenings of the meeting of the United States Veterinary Medical Association, at Des Moines, Iowa. The subjects to be discussed will be:

1. "Results of Prescribed Entrance Examination." Speaker: Dr. Wattles, Kansas City.

2. "State Boards of Veterinary Medical Examiners and Their Relation to Veterinary Colleges." Speaker: Dr. Detmers, Columbus, O.

3. "Uniform Course of Instruction in the Schools Belonging to the Association of Veterinary Faculties." Speaker: Dr. Adams, Philadelphia.

4. "Mutual Recognition of Students by Colleges Belonging to this Association." Speaker: Dr. Gill, New York.

5. "Uniform Veterinary Degree." Speaker: Prof. D. McEachran of Montreal and Dr. Schwarzkopf, Chicago.

6. "Competitive Examinations for Positions in Veterinary Faculties." Speaker: Dr. Liautard, New York.

7. "College Fees."

The Chairman of Executive Committee is Olof Schwarzkopf, V.M.D., McKillip Veterinary College, Chicago.

